# Supplementary material for: Single-Cell RNA-Seq Analysis Reveals Lung Epithelial Cell Type-Specific Responses to HDM and Regulation by Tet1
Source: Genes (Basel). 2022 May 14;13(5):880. doi: 10.3390/genes13050880 (PMC9140484; doi:10.3390/genes13050880)
Supplement: Supplementary file 1 [file genes-13-00880-s001.zip › Supplementary Table S2-Cell number in each group identified by scRNA-seq analysis.pdf]

Supplementary Table S2. Cell number in each group identified by scRNA-seq analysis

	WT-Sal	WT-HDM	KO-Sal	KO-HDM	Total
$EpCAM^+$ cells	6378	6925	5420	6714	25437
AT1 cells	18 (0.282%)	5 (0.072%)	9 (0.166%)	8 (0.119%)	40 (0.157%)
AT2 cells	5502 (86.265%)	3885 (56.101%)	4572 (84.354%)	3770 (56.151%)	17729 (69.698%)
Alveolar progenitor cells	157 (2.462%)	1758 (25.386%)	84 (1.550%)	1905 (28.374%)	3904 (15.348%)
Broncho alveolar stem cells	55 (0.862%)	985 (14.224%)	62 (1.144%)	671 (9.994%)	1773 (6.970%)
Ciliated cells	362 (5.676%)	66 (0.953%)	189 (3.487%)	131 (1.951%)	748 (2.941%)
Stromal cells	105 (1.646%)	88 (1.271%)	354 (6.531%)	38 (0.566%)	585 (2.300%)
Club cells	95 (1.489%)	75 (1.083%)	115 (2.122%)	139 (2.070%)	424 (1.667%)
Goblet cells	5 (0.078%)	56 (0.809%)	20 (0.369%)	45 (0.670%)	126 (0.495%)
Neuroendocrine cells	79 (1.239%)	7 (0.101%)	15 (0.277%)	7 (0.104%)	108 (0.425%)
