# Supplementary figures and images for: Single-Cell RNA-Seq Analysis Reveals Lung Epithelial Cell Type-Specific Responses to HDM and Regulation by Tet1
Source: Genes (Basel). 2022 May 14;13(5):880. doi: 10.3390/genes13050880 (PMC9140484; doi:10.3390/genes13050880)

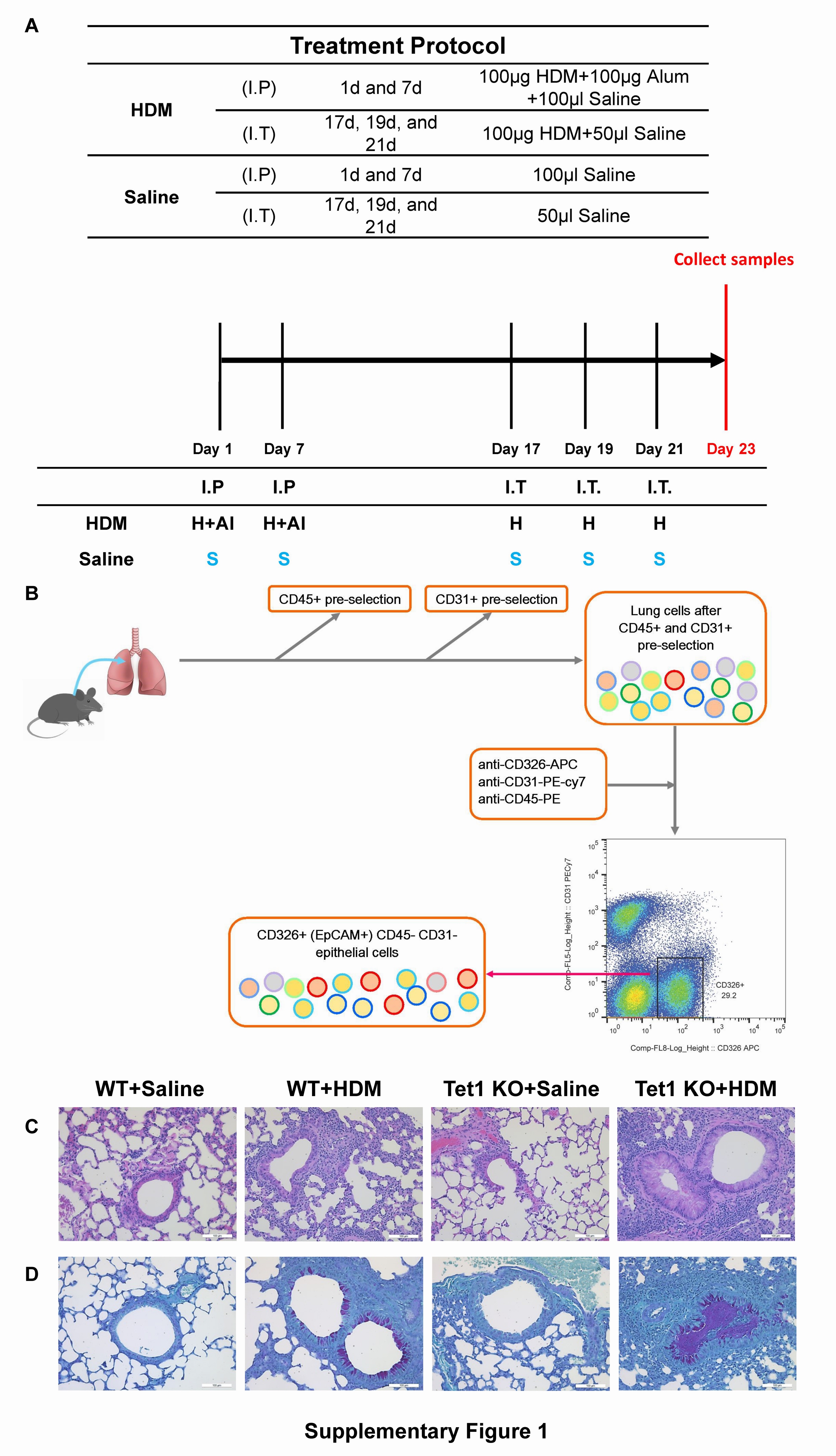

Supplement: Supplementary file 1 [file genes-13-00880-s001.zip › Supplementary Figure S1.jpg]

**Epcam, All Cells**

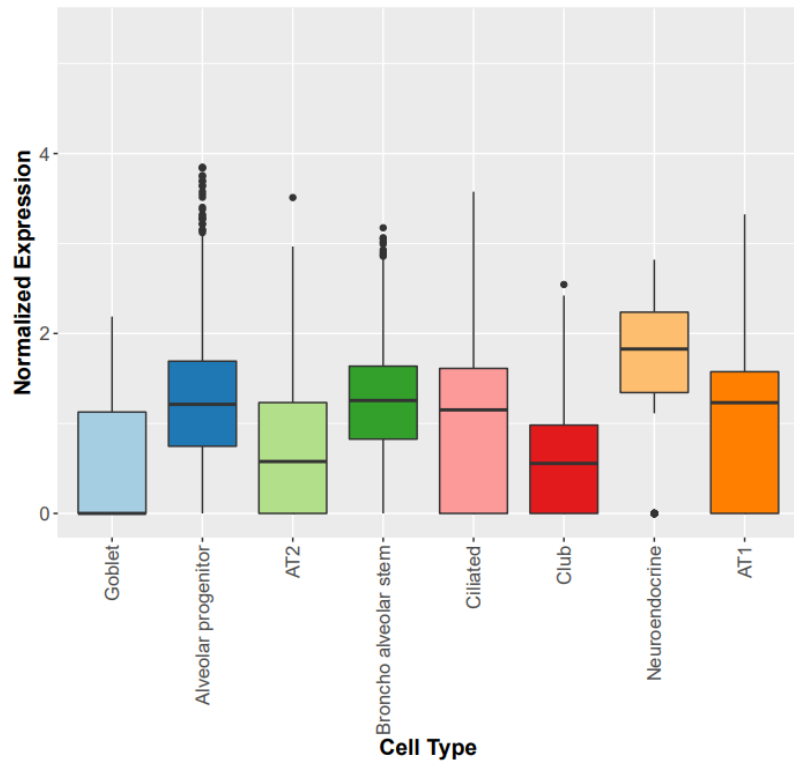

**Scgb1a1, All Cells**

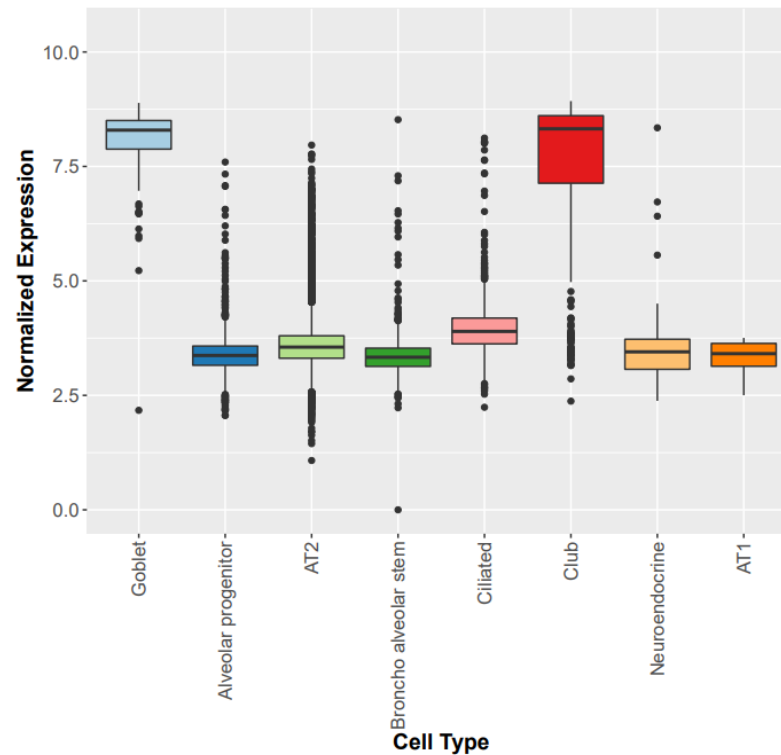

**Tet1, All Cells**

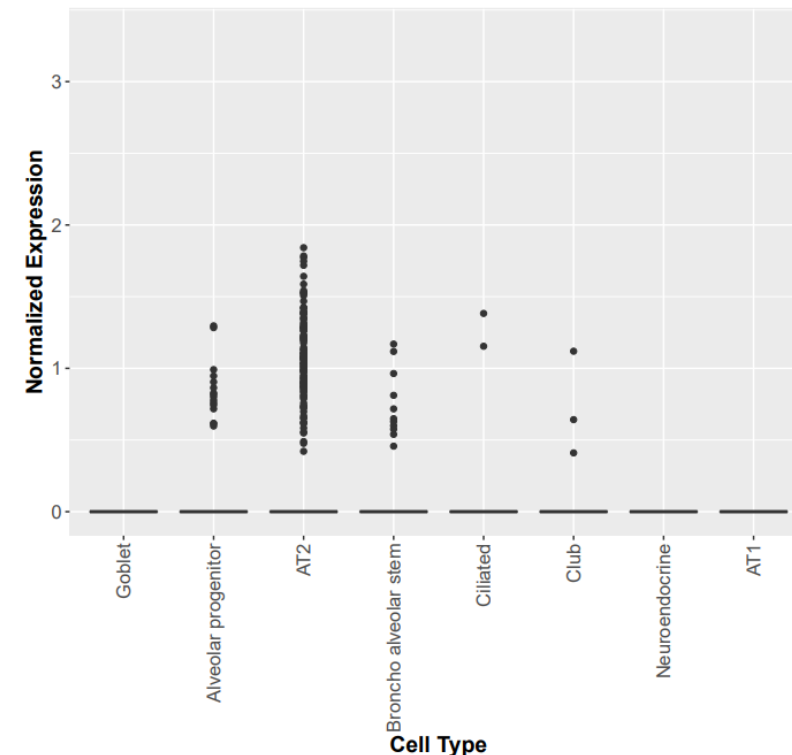

**Supplementary Figure S2**

Supplement: Supplementary file 1 [file genes-13-00880-s001.zip › Supplementary Figure S2_R1.pdf]

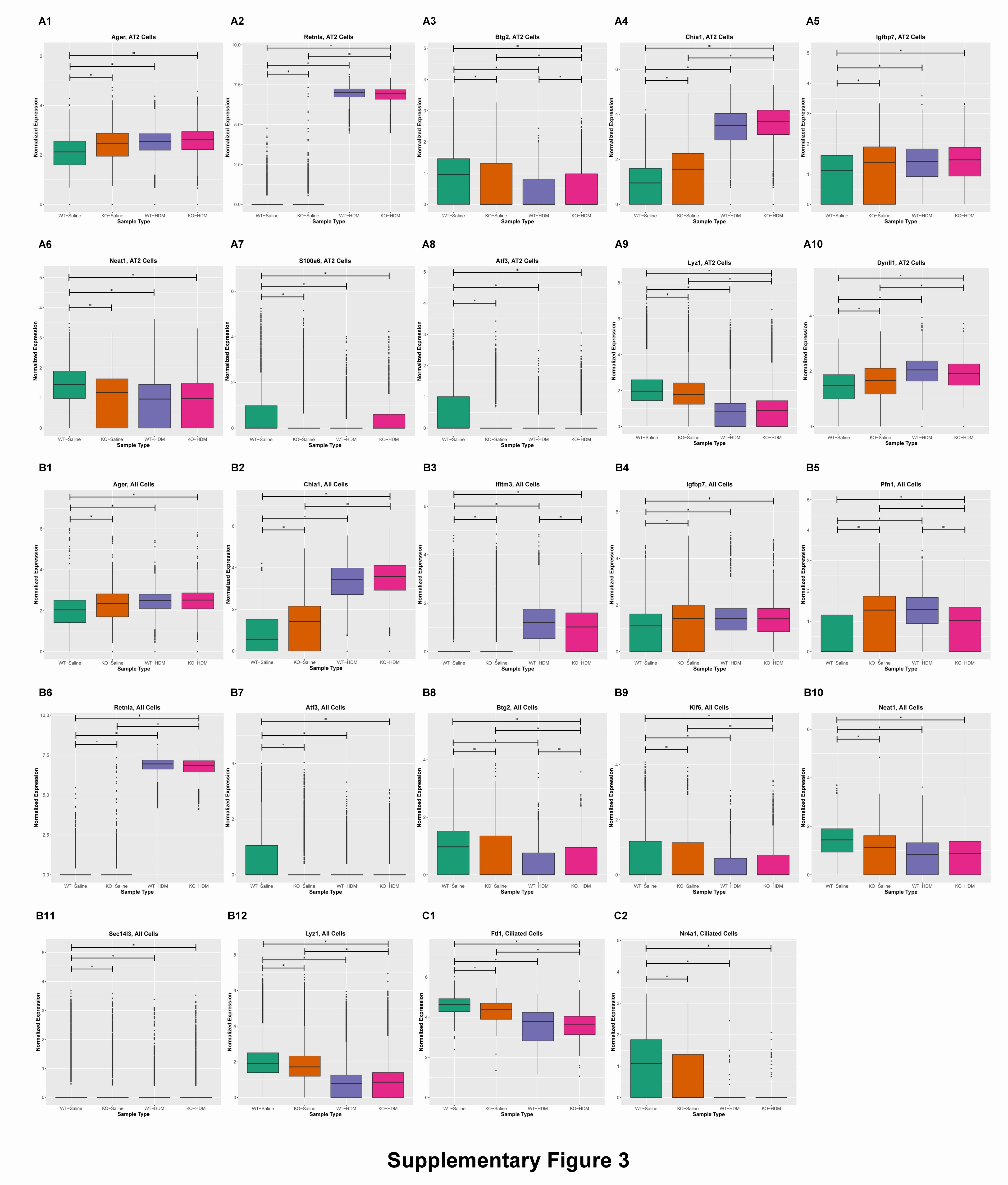

Supplement: Supplementary file 1 [file genes-13-00880-s001.zip › Supplementary Figure S3.jpg]

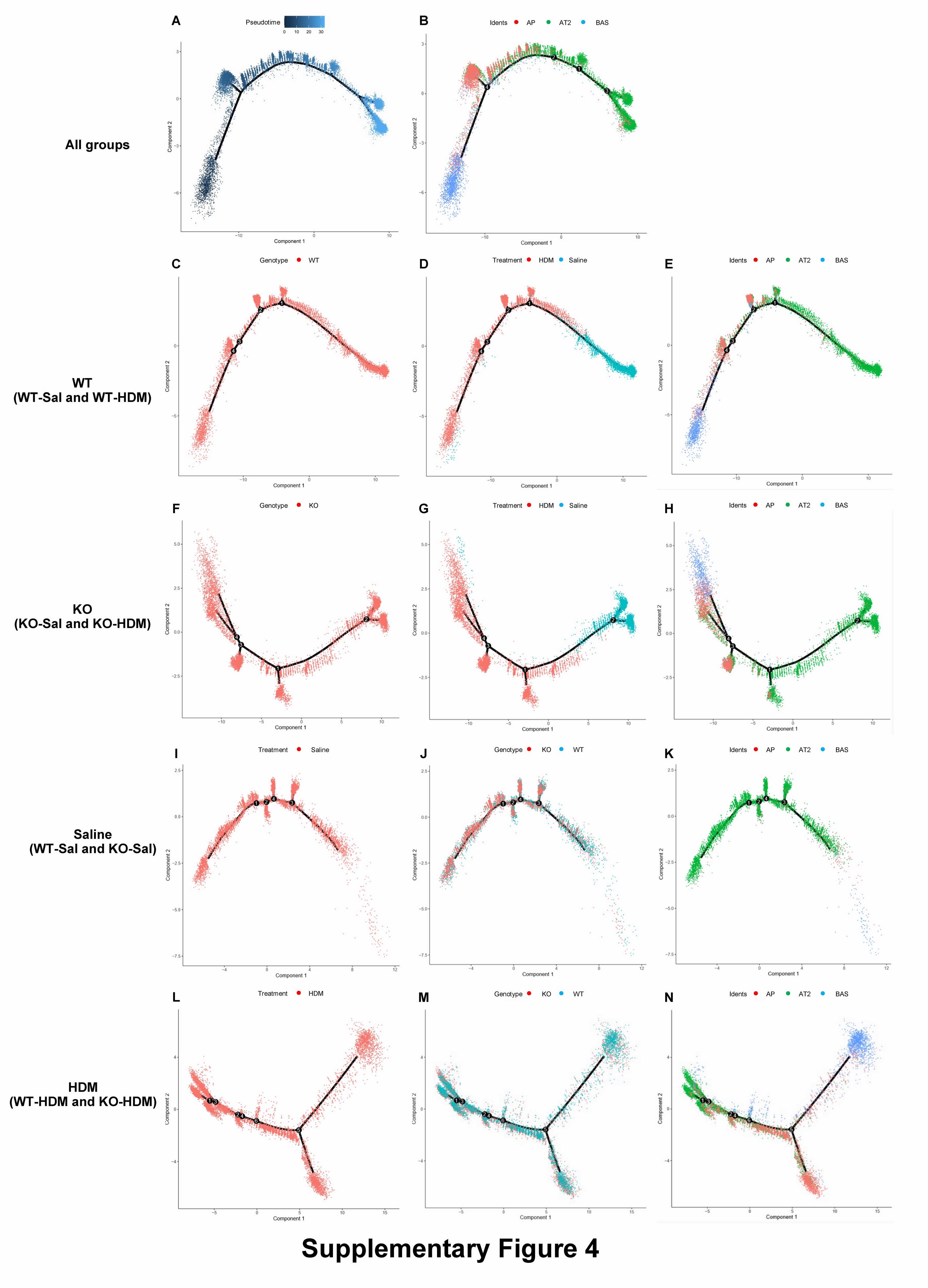

Supplement: Supplementary file 1 [file genes-13-00880-s001.zip › Supplementary Figure S4.jpg]

**A****All groups**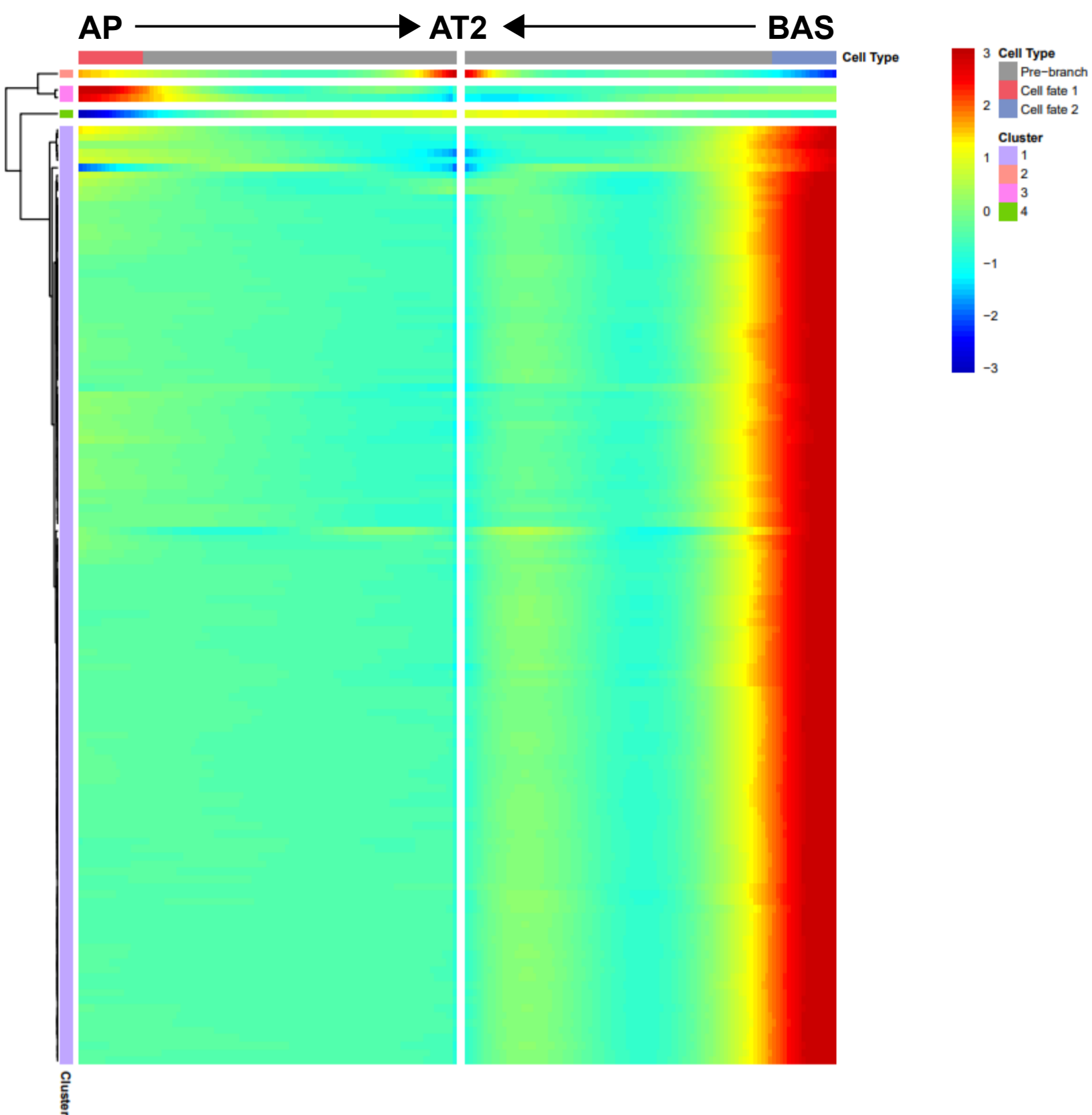**B****WT only  
(WT-Sal and WT-HDM)**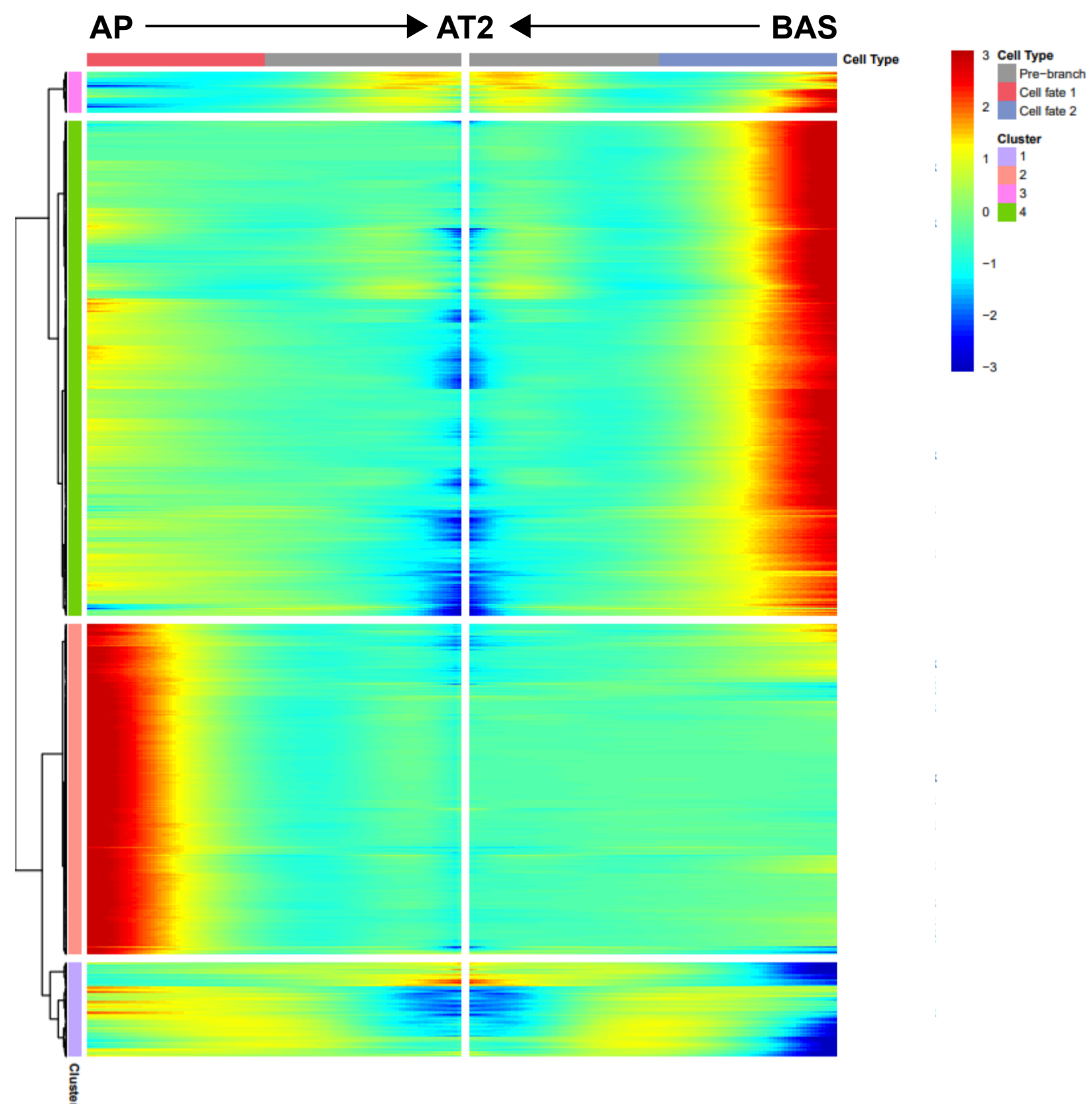**C****KO only  
(KO-Sal and KO-HDM)**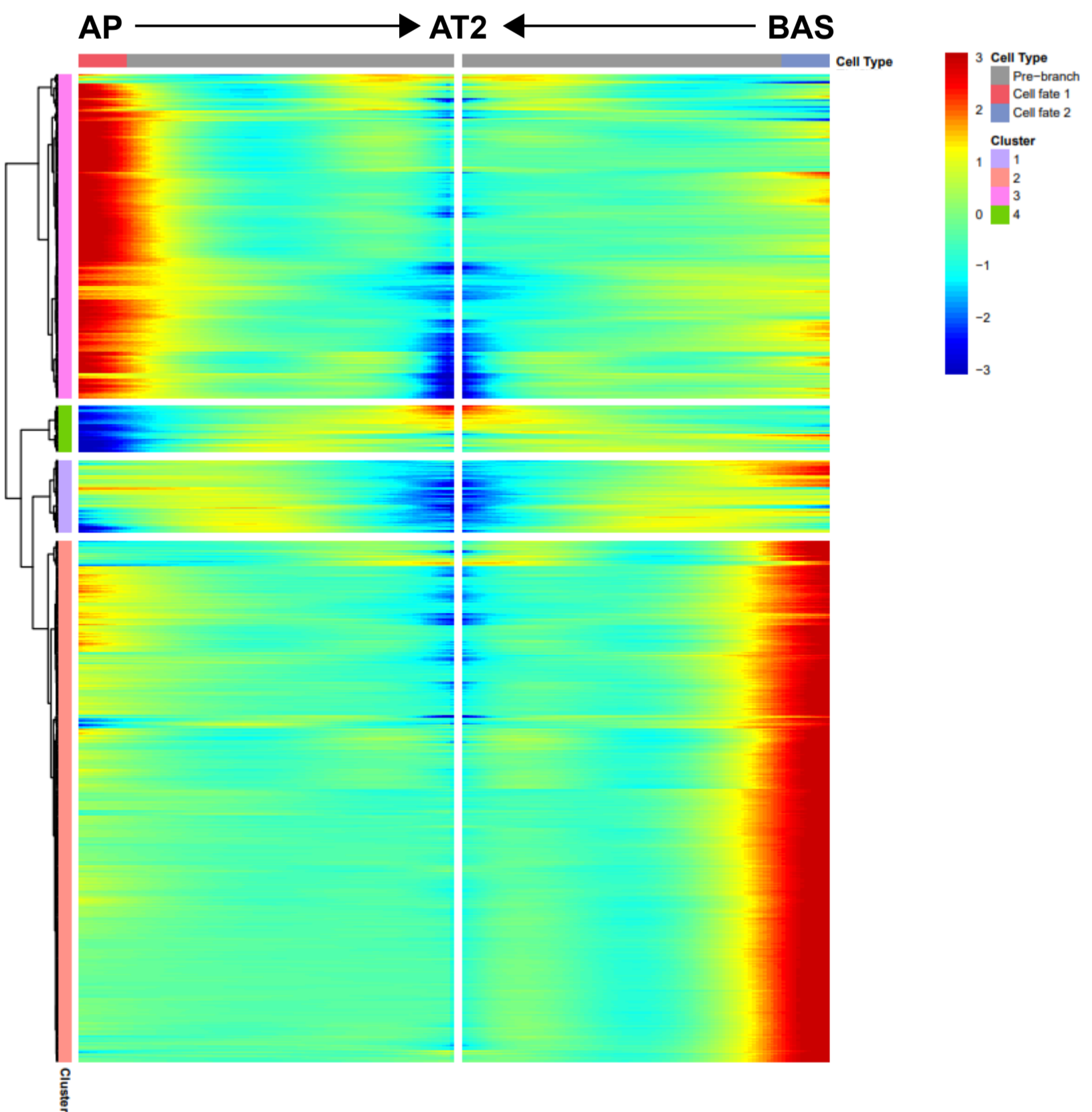**D****HDM only  
(WT-HDM and KO-HDM)**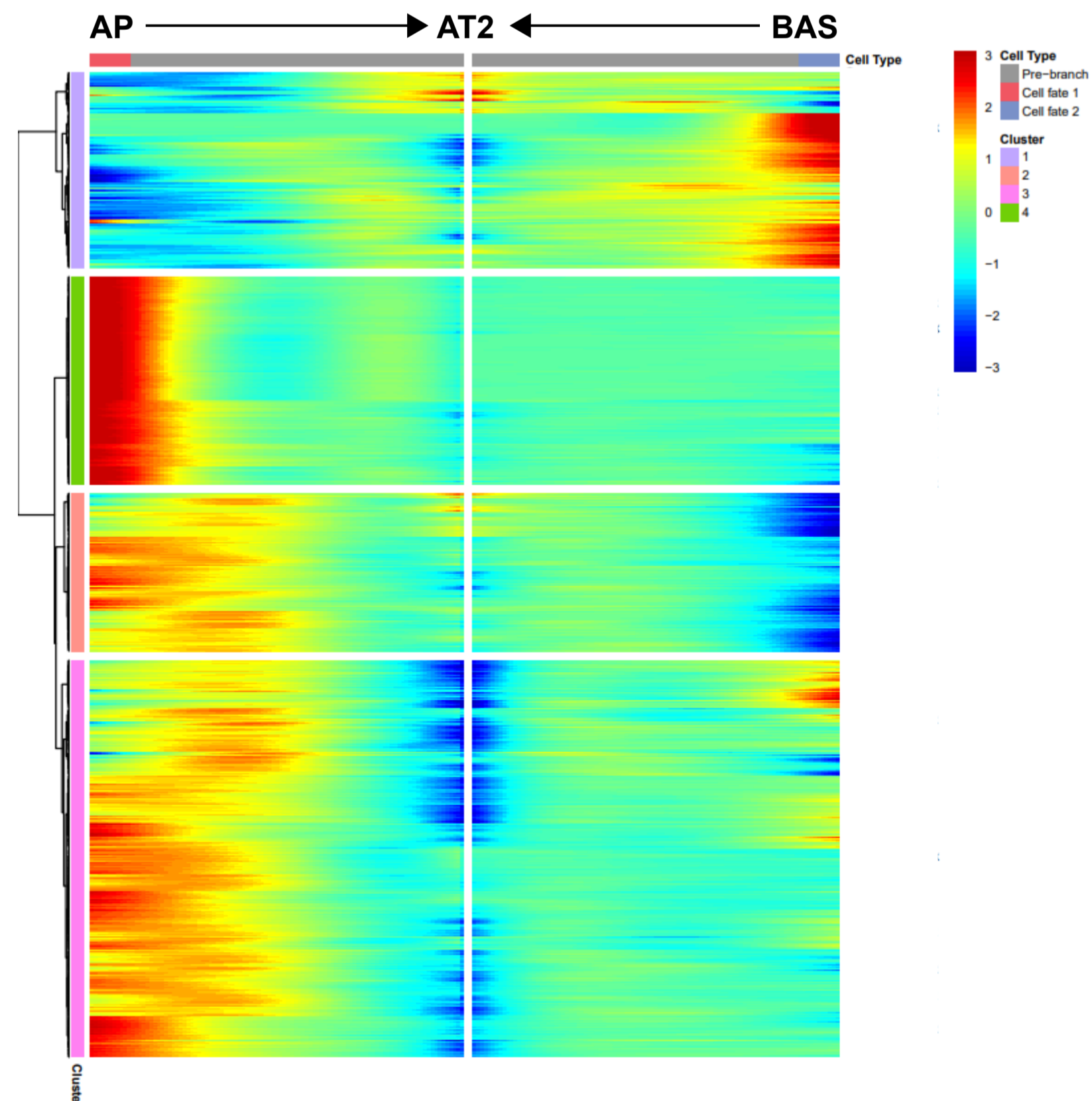

Supplement: Supplementary file 1 [file genes-13-00880-s001.zip › Supplementary Figure S5.pdf]

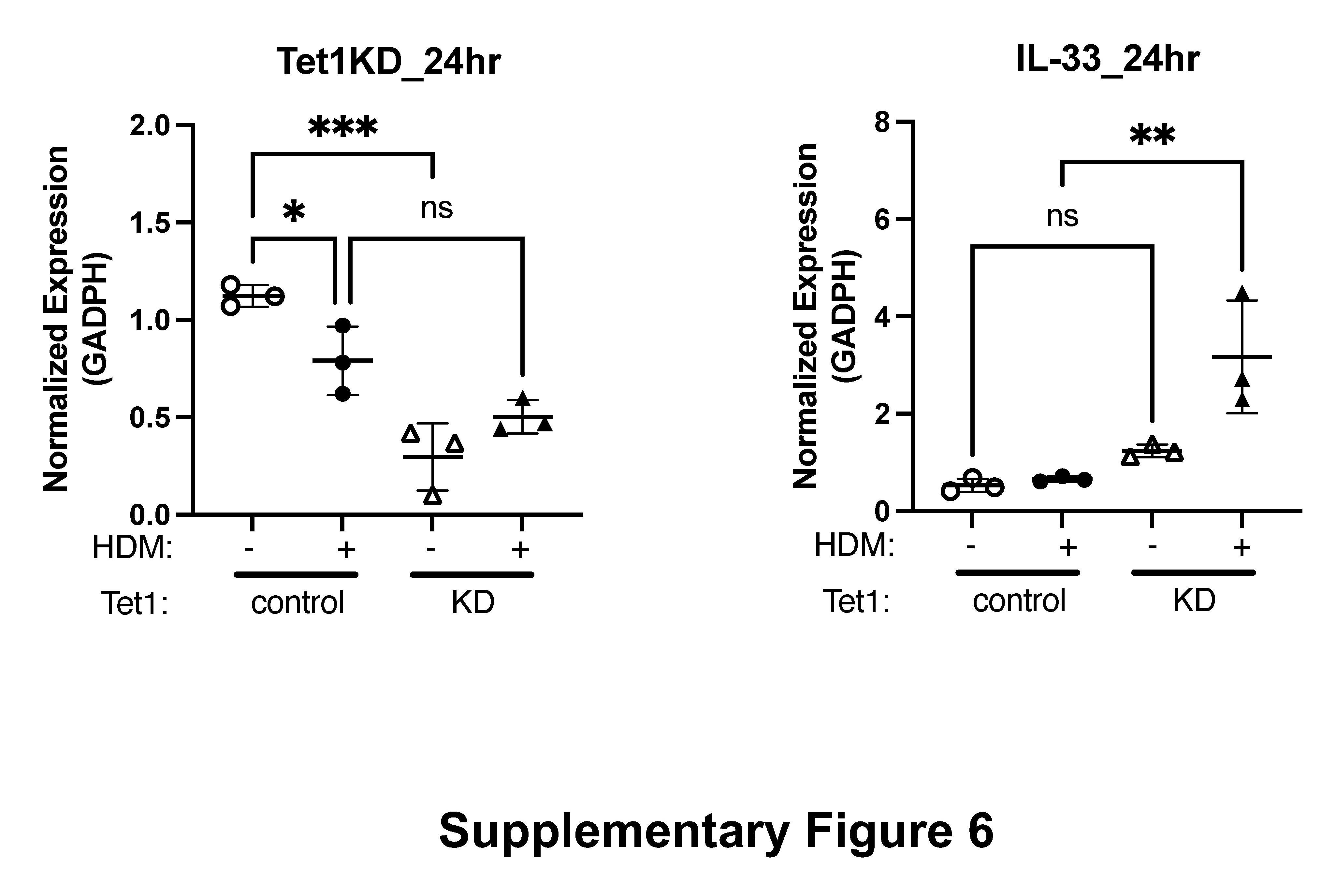

Supplement: Supplementary file 1 [file genes-13-00880-s001.zip › Supplementary Figure S6-1.jpg]
